# Differences in prevalence and management of chronic kidney disease among T2DM inpatients at the grassroots in Beijing and Taiyuan: a retrospective study

**DOI:** 10.1186/s41043-023-00406-1

**Published:** 2023-07-05

**Authors:** Lingwang An, Dandan Wang, Xiaorong Shi, Yali He, Yaujiunn Lee, Juming Lu

**Affiliations:** 1 Department of Endocrinology, Beijing Ruijing Diabetes Hospital, Beijing, 100079 China; 2 Department of Endocrinology, Taiyuan Diabetes Hospital, Taiyuan, 030013 China; 3 Department of Metabolism and Endocrinology, Lee’s Clinic, Pingtung, 90000 Taiwan China; 4 Department of Endocrinology, The General Hospital of the People’s Liberation Army, Ch No. 28 of Fuxing Road, Haidian District, Beijing, 100853 China

**Keywords:** Chronic kidney disease (CKD), Type 2 diabetes mellitus (T2DM), Dietary assessment, Regional differences

## Abstract

**Purpose:**

Chronic kidney disease (CKD) has been one of the most common complications in type 2 diabetes mellitus (T2DM) patients. This retrospective study aimed to investigate the regional differences in the prevalence and management of CKD in T2DM inpatients from two grassroots hospitals in Beijing and Taiyuan.

**Methods:**

The sociodemographic status, health history, lifestyle information, biochemical parameters and drug choices of the patients were collected from the Diabetes Care Information System using a retrospective cross-sectional analysis. The presence of CKD was defined as albuminuria (urine albumin-to-creatinine ratio of ≥ 30 mg/g) and/or as a reduced estimated glomerular filtration rate (< 60 ml/min/1.73 m^2^).

**Results:**

858 patients with T2DM in Beijing and 1,085 patients with T2DM in Taiyuan were included, with a median age of 61.0 and 61.9 years, respectively. The duration of diabetes was 10.5 and 10.3 years, respectively. The prevalence of CKD in Beijing (39.2%) was significantly higher than in Taiyuan (22.4%). The overall ABC control (A = haemoglobin A_1c_; B = blood pressure; C = cholesterol) in both the Beijing and Taiyuan groups were not ideal. Patients with CKD tended to use insulin, renin–angiotensin–aldosterone system (RAAS) inhibitors, sodium-glucose cotransporter-2 inhibitors (SGLT-2i) and dyslipidaemia therapy in Taiyuan than in Beijing. The actual proportion of carbohydrate, fat and protein in calories was 49.6%:35.4%:14.4% in Beijing and 61.5%:27.8%:10.8% in Taiyuan.

**Conclusions:**

The higher prescription rates of RAAS inhibitors, SGLT-2i and dyslipidaemia therapy may underlie the fluctuations in the prevalence of CKD in Beijing or Taiyuan. Intensive insulin therapy and personal nutritional guidance, along with the extensive use of RAAS inhibitors, SGLT-2i and dyslipidaemia therapy during follow-up, can all play a positive role in the management of CKD in patients with T2DM in both Beijing and Taiyuan.

**Supplementary Information:**

The online version contains supplementary material available at 10.1186/s41043-023-00406-1.

## Introduction

The increasing prevalence of diabetes has led to a worldwide rise in chronic kidney disease (CKD) over the last two decades. The microvascular complications in type 2 diabetes mellitus (T2DM) like diabetic nephropathy and diabetic retinopathy has been detected in more and more T2DM patients [1]. According to recent reports, diabetes was the third main cause of CKD onset, progression and development of end-stage renal disease [2, 3]. Although, in Chinese patients with T2DM, the prevalence of CKD ranged from 31.6% to 52.3% in different studies [4–6], the highest prevalence (52.3%) was among hospitalised patients. This finding is like that of a Singapore report (53%) taken from the diabetes mellitus centre of an acute care public hospital/primary care polyclinic [6, 7].

The modifiable risk factors for CKD onset and progression in patients with T2DM have been intensively studied and were discussed in our previous report without diet and geographic disparities [3]. They are comprised of alcohol and tobacco use, levels of physical activity, issues of overweight or obesity and ABC control (A, haemoglobin A_1c_ [HbA_1c_]; B, blood pressure [BP]; and C, cholesterol) [8]. The ABC control in Chinese T2DM inpatients with CKD was poor, with relatively high HbA_1c_, systolic BP (SBP), diastolic BP (DBP), increased low density lipoprotein C (LDL-C) and triglycerides (TG) [4, 6]. Diet was also a modifiable risk factor for CKD. A higher total consumption of red and/or processed meat was associated with an increased risk of incident CKD, while plant protein intake was associated with a decrease in the risk of incident CKD [9, 10].

Geographic disparities in CKD prevalence have been reported [11–13], with similar risk factors—smoking, physical activity, dietary preferences, socioeconomic status, etc.—and laboratory methods contributing to the differences. However, few Chinese studies have examined the prevalence and management of CKD in hospitalised T2DM patients from different regions at the same time.

The Beijing Ruijing Diabetes Hospital and the Taiyuan Diabetes Hospital are primary chain hospitals in China. Patients have been included in multi-disciplinary management, screenings for diabetic complications as well as nutritional assessment and guidance since 2016. Information systems were developed for the recording of nutritional assessment and guidance in 2018. This study aimed to evaluate the prevalence of CKD in hospitalised patients with T2DM in these two regional hospitals. The associated modifiable metabolic risk factor controls, medication usage and dietary assessments and guidance were also investigated.

## Materials and methods

### Materials

#### Participants

During the inclusion period—lasting from 28 March, 2018, until 5 November, 2021—comprehensive information was collected from 2648 patients. Out of 2648 patients, 1943 were included and rest were excluded as they did not met the inclusion criteria of the study. Among them were 858 patients with T2DM in Beijing and 1085 in Taiyuan. This study followed the protocols defined by the declaration of Helsinki and was approved by the ethics committee of Beijing Ruijing Diabetes Hospital.

#### Inclusion criteria

1. Hospitalised patients with T2DM—ranging in age from 18 to 79 years.
2. Patients had records of HbA_1c_, estimated glomerular filtration rate (eGFR), urine albumin-to-creatinine ratio (UACR), SBP, DBP, LDL-C, TG and body mass index (BMI) testing and baseline measurements.
3. Patients had received nutritional assessments and guidance.

#### Exclusion criteria

1. Patients younger than 18 years old at the time of a diabetes diagnosis or with a fasting plasma C peptide of < 100 pmol/L for the possibility of other types of diabetes
2. Patients who have had serious comorbidities or uncontrolled conditions in their medical histories, including: malignant tumours, blindness, dialysis, diabetic foot ulcers, heart failure, liver disease, thyroid disease, an SBP of ≥ 190 mmHg or a DBP of ≥ 120 mmHg, haemoglobin < 90 g/dL, fasting plasma glucose (FPG) < 3.9 mmol/L, BMI < 18.5 kg/m^2^ or eGFR < 30.0 mL/min / 1.73 m^2^
3. Patients with incomplete nutritional assessments, an actual energy intake of < 600 kcal/day or an actual carbohydrate intake of < 50 g/day (ketogenic diet)

#### Study design

This was a retrospective cross-sectional study based on medical records included in the Diabetes Sharecare Information System (DSIS). Upon giving oral consent, patients would be registered within DSIS and involved in both comprehensive risk factor assessments and multi-disciplinary management [3]. Using a definitive food group model, along with a dietary review method, dietary assessments were conducted by trained dieticians during face-to-face interviews and stored in the system. Food portion sizes were reported using household measurements and then converted to their equivalents in grams and calories. The following food groups were identified: vegetables, fruits, cereals and tubers (combined), legumes, dairy products, meat and eggs (combined), nuts, oil and alcohol.

#### Setting

This analysis was supported by the DSIS baseline data of Beijing Ruijing Diabetes Hospital and Taiyuan Diabetes Hospital.

#### Study variables

Health history of hypertension, dyslipidemia, cardiovascular disease (angina, acute myocardial infarction, percutaneous coronary stent implantation and bypass, heart failure), cerebrovascular diseases (stroke/transient ischemic attack), retinopathy and diabetic kidney disease were collected. Other collected variables included socio-demographic status (age, gender, education and medical care), lifestyle (smoking, drinking and exercise patterns), BMI, anthropometric measurements (height, weight and resting BP), biochemical parameters (serum creatinine [SCr], haemoglobin, FPG, HbA_1c_, LDL-C, TG and UACR), medications, nutritional assessments and recommendations (actual and recommended daily intake of calories, carbohydrates, fats and protein).

#### Determination of biochemical parameters

The HbA_1c_ measurements were tested with high-performance liquid chromatography equipment. Biochemical parameters were evaluated by automatic biochemical analysers. The biochemical measurements were in full compliance with all local internal quality control standards.

#### CKD classification

GFR is the gold standard for renal function. Since the detection of GFR is too cumbersome, the Creatinine-based eGFR is still widely accepted in current clinical practice. Thus, the simplified Modification of Diet in Renal Disease (MDRD) equation for the Chinese study was used to calculate the eGFR based on SCr: MDRD = 175 × SCr (mg/dl)^−1.234^ × years^−0.179^ × (0.79, if female) [14]. The presence of CKD was defined as albuminuria (a UACR of ≥ 30 mg/g) and/or reduced eGFR (< 60 ml/min/1.73 m^2^) when conducting the nutritional assessment [15–18]. We defined CKD with the baseline eGFR and/or UACR measurement, as we were unable to ascertain if the above abnormalities persisted for more than three months. The diagnosis of CKD was classified based upon an eGFR category (G1–G5) and an albuminuria category (A1–A3) [3, 15–18].

#### BMI, blood lipid index, and hyperuricemia

The BMI was categorised either as normal: a BMI of 18.5–24.0 kg/m^2^, overweight: a BMI of 24.0–27.9 kg/m^2^, or obese: a BMI ≥ 28.0 kg/m^2^. The lipid targets were as follows: LDL-C < 2.6 mmol/L and TG < 1.7 mmol/L [19]. Hyperuricemia was defined as serum uric acid (SUA) > 420 μmol/L in male patients and > 360 μmol/L in female patients [20].

#### Ratio of carbohydrates, fats and protein

The ratio of carbohydrates, fats and protein can be defined as the energy derived from these three nutrient sources divided by the total daily energy expenditure [21] (A/R: actual/recommended; R‒A: recommended minus actual). Actual ratios of carbohydrate (< 50%, 50–60% and > 60%), fat (< 27%, 27–35% and > 35%), and protein intake (< 0.8 g, 0.8–1.0 g and > 1.0 g) per kilogram of ideal body weight (IBW) were categorised according to the third percentile. Actual ratio of protein (≤ 12% and > 12%) was categorised according to the median value.

#### Evaluation and assessment indicators

The primary outcome in Beijing and Taiyuan was the prevalence of CKD in hospitalised T2DM patients, as well as the importance of nutritional assessments. Secondary outcomes included the management of CKD patients with T2DM in Beijing and Taiyuan, including the control of modifiable metabolic risk factors, medication use, dietary habits and overall guidance.

### Statistical analysis

To better enunciate results, we renamed the groups as group 1 (T2D with CKD) and group 2 (T2D only). All analyses were performed using the SPSS 22.0 software. Categorical variables were expressed as numbered percentages (%). Continuous variables were expressed as the mean ± standard deviation for normally distributed variables, and as the median (25% and 75% quartile) for non-normally distributed variables. The differences in patient characteristics, risk factors, medications and complications were studied using a chi-square test for categorical variables, and the Kruskal–Wallis model for non-normally distributed continuous variables. Binary logistic regression was used to estimate the odds ratio (OR) for factors associated with CKD. A two-sided *P* value of < 0.05 was statistically significant.

## Results

### Comparison of the baseline data

Of the 858 adults with T2DM in Beijing and the 1,085 adults with T2DM in Taiyuan, the median age was 61.0 years and 61.9 years, respectively. The duration of diabetes was 10.5 years and 10.3 years, respectively. The demographic and clinical characteristics of the Beijing and Taiyuan individuals are shown in Table 1. The male patients numbered 58.6% in Beijing and 51.0% in Taiyuan. Compared with group 2, a significantly greater number of patients in group 1 had histories of hypertension, retinopathy, and diabetic kidney disease (DKD) both in Beijing and Taiyuan. Compared with group 1 in Beijing, a significantly greater number of patients in group 1 in Taiyuan had histories of retinopathy and DKD.

**Table 1:** Characteristics of patients with T2DM and CKD and T2DM only in Beijing and Taiyuan

Characteristics	Beijing				Taiyuan			
	Overall (n = 858)	Group 1 (n = 336)	Group 2 (n = 522)	*P*	Overall (n = 1085)	Group 1 (n = 243)	Group 2 (n = 842)	*P*
Age (years)	61.0 (53.6, 66.3)	61.7 (54.2, 66.8)	60.4 (53.4, 65.9)	0.121	61.9 (55.5, 67.2)^#^	62.2 (55.7, 68.3)	61.7 (55.5, 67.0)	0.378
Age (≥ 60 years)	471 (54.9)	197 (58.6)	274 (52.5)	0.078	639 (58.9)	145 (59.7)	494 (58.7)	0.780
Gender (male)	503 (58.6)	213 (63.4)	290 (55.6)	0.023	553 (51.0)^#^	137 (56.4)	416 (49.4)	0.055
Diabetes duration (years)	10.5 (4.7, 16.8)	11.0 (6.7, 20.1)	9.7 (3.7, 15.3)	< 0.001	10.3 (4.6, 14.8)	12.4 (7.3, 19.0)	9.0 (3.8, 13.6)	< 0.001
< 5.0	228 (26.6)	63 (18.8)	165 (31.6)	< 0.001	286 (26.4)^#^	40 (16.5) *	246 (29.2)	< 0.001
5.0–9.9	161 (18.8)	62 (18.5)	99 (19.0)		231 (21.3)	33 (13.6)	198 (23.5)	
10.0–14.9	195 (22.7)	72 (21.4)	123 (23.6)		306 (28.2)	76 (31.3)	230 (27.3)	
≥ 15.0	274 (31.9)	139 (41.4)	135 (25.9)		262 (24.1)	94 (38.7)	168 (20.0)	
Education								
Below high school	548 (65.0)	227 (68.4)	321 (62.8)	0.098	643 (59.3) *	154 (63.6)	489 (58.1)	0.121
Above high school	295 (35.0)	105 (31.6)	190 (37.2)		441 (40.7)	88 (36.4)	353 (41.9)	
Comorbidities/complications								
Hypertension	483 (56.3)	226 (67.3)	257 (49.2)	< 0.001	567 (52.3)	166 (68.3)	401 (47.6)	< 0.001
Dyslipidemia	530 (61.8)	221 (65.8)	309 (59.2)	0.053	390 (35.9)^#^	88 (36.2) ^#^	302 (35.9)	0.921
Cardiovascular disease	229 (26.7)	101 (30.1)	128 (24.5)	0.073	269 (24.8)	70 (28.8)	199 (23.6)	0.100
Cerebrovascular diseases	105 (12.2)	43 (12.8)	62 (11.9)	0.688	114 (10.5)	31 (12.8)	83 (9.9)	0.194
Retinopathy	101 (11.8)	55 (16.4)	46 (8.8)	0.001	272 (25.1)^#^	121 (49.8) ^#^	151 (17.9)	< 0.001
Diabetic kidney disease	114 (13.3)	100 (29.8)	14 (2.7)	< 0.001	153 (14.1)	130 (53.5) ^#^	23 (2.7)	< 0.001

CKD: chronic kidney disease; Beijing vs. Taiyuan,*P < 0.05, ^#^*P* < 0.001. Categorical variables were expressed as numbers (%). Continuous variables were expressed as median (25% and 75% quartile) for non-normally distributed variables

Group 1: T2DM and CKD Group 1: T2DM only

### The prevalence of CKD in Beijing and Taiyuan

The prevalence of CKD in Beijing was 39.2% (336), which was significantly higher than the 22.4% (243) in Taiyuan. The prevalence of reduced eGFR (with or without albuminuria) and albuminuria (with or without reduced eGFR) was 2.9% and 38.6% in Beijing and 2.6% and 21.6% in Taiyuan, respectively. The prevalence of microalbuminuria and macroalbuminuria was 30.3% and 8.3% in Beijing and 17.8% and 3.8% in Taiyuan, respectively (Additional file 1: Table S1).

### Follow-up management of CKD in Beijing and Taiyuan

Follow-up management protocols are different in the cases of Taiyuan and Beijing. In both Beijing and Taiyuan, the median HbA_1c_, SBP, hyperuricemia, and insulin usage (both current and follow-up medication) in group 1 were significantly higher than in group 2 (*P* < 0.001). In both Beijing and Taiyuan, a significantly greater number of patients in group 1 had a HbA_1c_ of ≥ 9.0%, a BP of ≥ 130/80 mmHg and hyperuricemia. In group 1 of Beijing, the only significant increase was in the number of patients who had a TG of ≥ 1.7 mmol/L and who were obese. At baseline, significantly more patients in group 1 of Taiyuan used sodium-glucose cotransporter-2 inhibitors (SGLT-2i), angiotensin-converting enzyme inhibitors (ACEI)/angiotensin receptor blockers (ARB), statins and dyslipidaemia therapy than in group 1 of Beijing. However, during follow-up management, the usage rate increased significantly, with a greater number of patients using SGLT-2i, ACEI/ARB, statins and dyslipidaemia therapy in both group 1 in the Beijing and Taiyuan (Table 2).

**Table 2:** Risk factors control and medications in T2DM and CKD group and T2DM only group of Beijing and Taiyuan

Parameters	Beijing				Taiyuan			
	overall (n = 858)	Group 1 (n = 336)	Group 2 (n = 522)	*P*	overall (n = 1085)	Group 1 (n = 243)	Group 2 (n = 842)	*P*
HbA_1c_ (%)	8.4 (7.1, 9.9)	8.7 (7.5, 10.1)	8.1 (7.0, 9.7)	< 0.001	8.5 (7.2, 10.1)	9.2 (7.9, 10.8) #	8.2 (7.1, 10.0)	< 0.001
< 7.0	183 (21.3)	57 (17.0)	126 (24.1)	0.002	214 (19.7)	23 (9.5) *	191 (22.7)	< 0.001
7.0–8.9	342 (39.9)	125 (37.2)	217 (41.6)		424 (39.1)	86 (35.4)	338 (40.1)	
≥ 9.0	333 (38.8)	154 (45.8)	179 (34.3)		447 (41.2)	134 (55.1)	313 (37.2)	
SBP (mmHg)	131 (120, 147)	136 (125, 150)	130 (120, 142)	< 0.001	130 (120, 140) #	138 (125, 150)	130 (120, 140)	< 0.001
DBP (mmHg)	80 (72, 87)	80 (71, 90)	80 (72, 86)	0.252	80 (70, 86) #	80 (71, 88)	79 (70, 85)	0.007
BP ≥ 130/80 mmHg	616 (71.8)	260 (77.4)	356 (68.2)	0.004	739 (68.1)	188 (77.4)	551 (65.4)	< 0.001
LDL-C (mmol/L)	2.93 (2.32, 3.55)	2.92 (2.39, 3.56)	2.94 (2.26, 3.54)	0.549	2.83 (2.24, 3.46) #	2.96 (2.32, 3.57)	2.80 (2.23, 3.39)	0.045
LDL-C ≥ 2.6 mmol/L	551 (64.2)	214 (63.7)	337 (64.6)	0.796	661 (60.9)	157 (64.6)	504 (59.9)	0.181
TG (mmol/L)	1.63 (1.13, 2.43)	1.79 (1.19, 2.74)	1.54 (1.11, 2.23)	0.001	1.73 (1.24, 2.50) *	1.87 (1.30, 2.92)	1.69 (1.21, 2.38)	0.003
TG ≥ 1.7 mmol/L	410 (47.8)	183 (54.5)	227 (43.5)	0.002	556 (51.2)	136 (56.0)	420 (49.9)	0.095
Hyperuricemia	250 (29.2)	124 (36.9)	126 (24.3)	< 0.001	203 (18.8) #	70 (28.8) *	133 (15.9)	< 0.001
BMI (kg/m2)	26.1 (23.9, 28.4)	26.6 (24.2, 28.9)	25.8 (23.7, 28.0)	0.001	25.1 (23.1, 27.3)	25.4 (23.5, 27.7) #	25.1 (23.1, 27.1)	0.158
Normal	219 (25.5)	75 (22.3)	144 (27.6)	0.003	364 (33.5) #	71 (29.2) #	293 (34.8)	0.253
Overweight	381 (44.4)	138 (41.1)	243 (46.6)		502 (46.3)	118 (48.6)	384 (45.6)	
Obesity	258 (30.1)	123 (36.6)	135 (25.9)		219 (20.2)	54 (22.2)	165 (19.6)	
Current smoker	247 (28.8)	106 (31.5)	141 (27.0)	0.152	192 (17.7) #	49 (20.2) #	143 (17.0)	0.252
Current drinker	179 (20.9)	78 (23.2)	101 (19.3)	0.174	145 (13.4) #	33 (13.6) #	112 (13.3)	0.910
No regular exercise	798 (94.5)	321 (96.4)	477 (93.3)	0.056	495 (45.7) #	127 (52.5) #	368 (43.8)	0.017
Current medication								
Insulin usage	314 (36.6)	147 (43.8)	167 (32.0)	< 0.001	504 (46.5) #	155 (63.8) #	349 (41.4)	< 0.001
SGLT-2i usage	29 (3.4)	10 (3.0)	19 (3.6)	0.600	142 (13.1) #	41 (16.9) #	101 (12.0)	0.047
ACEI/ARB usage	194 (22.6)	84 (25.0)	110 (21.1)	0.180	405 (37.3) #	147 (60.5) #	258 (30.6)	< 0.001
Statins usage	230 (26.8)	94 (28.0)	136 (26.1)	0.535	697 (64.2) #	170 (70.0) #	527 (62.6)	0.035
Dyslipidemia therapy	232 (27.0)	96 (28.6)	136 (26.1)	0.418	743 (68.5) #	186 (76.5) #	557 (66.2)	0.002
Follow up medication								
Insulin usage	511 (59.6)	233 (69.3)	278 (53.3)	< 0.001	528 (48.7) #	164 (67.5)	364 (43.2)	< 0.001
SGLT-2i usage	430 (50.1)	186 (55.4)	244 (46.7)	0.014	200 (18.4) #	56 (23.0)	144 (17.1)	0.035
ACEI/ARB usage	393 (45.8)	181 (53.9)	212 (40.6)	< 0.001	437 (40.3) *	157 (64.6)	280 (33.3)	< 0.001
Statins usage	625 (72.8)	258 (76.8)	367 (70.3)	0.037	719 (66.3) #	178 (73.3)	541 (64.3)	0.009
Dyslipidemia therapy	639 (74.5)	264 (78.6)	375 (71.8)	0.027	764 (70.4) *	191 (78.6)	573 (68.1)	0.002

CKD: Chronic kidney disease; SBP: systolic blood pressure; DBP: diastolic blood pressure; LDL-C: low density lipoprotein cholesterol; TG: triglycerides; BMI: body mass index; SGLT-2i: sodium-glucose cotransporter-2 inhibitors; ACEI: angiotensin converting enzyme inhibitors; ARB: angiotensin receptor antagonist; Beijing vs. Taiyuan,*P < 0.05, #*P* < 0.001. Categorical variables were expressed as numbers (%). Continuous variables were expressed as median (25% and 75% quartile) for non-normally distributed variables

Group 1: T2DM and CKD Group 1: T2DM only

### Dietary assessments and recommendations in Beijing and Taiyuan

There are differences in the dietary structures of the patients in Beijing and Taiyuan. In general, actual fat intake was higher in Beijing (A/R: 123.3% [92.5–164.3%]), carbohydrate intake was higher in Taiyuan (A/R: 116.2% [95.0–144.0%]) and protein intake was lower in both Beijing (A/R: 92.1% [70.7–115.3%]) and Taiyuan (A/R: 64.6% [52.3–84.1%]). When comparing Taiyuan to Beijing, significantly more patients had an actual carbohydrate ratio of > 60% and an actual fat ratio of < 27%. In Taiyuan, the recommended carbohydrate and fat ratio was higher in group 1 than group 2, while the protein ratio was lower. In both Beijing and Taiyuan, there was a significant difference in the recommended protein intake of 0.8, 0.8–1.0, and > 1.0 g per kilogram of IBW between group 1 and group 2 (Additional file 1: Table S2).

### The risk factors associated with CKD incidence

After controlling for potential sources of error, the following factors were identified as being associated with an increased risk of CKD in the overall study population: male, a diabetic duration of ≥ 10 years, an HbA_1c_ of ≥ 9.0%, uncontrolled BP (≥ 130/80 mmHg) and TG (≥ 1.7 mmol/L), a lack of regular physical activity, a history of hypertension, retinopathy, DKD and hyperuricemia. In the Beijing participants, a fat ratio of < 27% was associated with a lower risk of CKD, along with an OR of 0.43 (95% confidence interval: 0.19–0.96), while in the Taiyuan participants, there were no additional relevant factors (Table 3).

**Table 3:** CKD associated factors in Beijing and Taiyuan

Parameters	Overall		Beijing		Taiyuan	
	*P*	OR (95% CI)	*P*	OR (95% CI)	*P*	OR (95% CI)
Age (≥ 60 vs < 60 years)	0.234	1.18 (0.90–1.55)	0.138	1.32 (0.91–1.92)	0.792	1.06 (0.69–1.61)
Male vs female	0.044	1.34 (1.01–1.78)	0.121	1.36 (0.92–2.00)	0.219	1.33 (0.84–2.11)
*Diabetes duration (years)*						
< 5.0		Reference		Reference		Reference
5.0–9.9	0.341	1.20 (0.82–1.76)	0.108	1.51 (0.91–2.50)	0.542	0.82 (0.43–1.55)
10.0–14.9	0.010	1.60 (1.12–2.28)	0.122	1.47 (0.90–2.38)	0.053	1.73 (0.99–3.00)
≥ 15.0	0.000	2.43 (1.70–3.46)	0.000	2.42 (1.51–3.87)	0.025	1.94 (1.09–3.44)
Education (below vs above high school)	0.075	1.27 (0.98–1.66)	0.686	1.08 (0.75–1.55)	0.122	1.40 (0.92–2.13)
*BMI category*						
Normal		Reference		Reference		Reference
Overweight	0.990	1.00 (0.75–1.34)	0.687	0.92 (0.61–1.38)	0.948	1.02 (0.64–1.60)
Obesity	0.304	1.20 (0.85–1.68)	0.508	1.17 (0.74–1.84)	0.813	0.94 (0.53–1.64)
*HbA*_*1c*_* (%)*						
< 7.0		Reference		Reference		Reference
7.0–8.9	0.051	1.45 (1.00–2.09)	0.147	1.41 (0.89–2.24)	0.109	1.72 (0.89–3.33)
≥ 9.0	0.000	2.61 (1.81–3.77)	0.000	2.33 (1.46–3.71)	0.000	4.00 (2.07–7.72)
BP ≥ 130/80 mmHg (yes vs no)	0.026	1.38 (1.04–1.83)	0.026	1.54 (1.05–2.25)	0.259	1.30 (0.83–2.04)
LDL-C ≥ 2.6 mmol/L (yes vs no)	0.781	0.96 (0.74–1.25)	0.256	0.81 (0.56–1.16)	0.576	1.12 (0.75–1.69)
TG ≥ 1.7 mmol/L (yes vs no)	0.037	1.32 (1.02–1.71)	0.042	1.45 (1.01–2.08)	0.089	1.43 (0.95–2.16)
Hyperuricemia (yes vs no)	0.000	1.72 (1.29–2.28)	0.142	1.31 (0.91–1.88)	0.000	2.47 (1.51–4.04)
Current smoker (yes vs no)	0.166	1.28 (0.90–1.80)	0.621	1.11 (0.73–1.70)	0.516	1.23 (0.66–2.27)
Current drinker (yes vs no)	0.445	0.86 (0.59–1.26)	0.762	0.93 (0.59–1.47)	0.499	0.79 (0.40–1.57)
No regular exercise (yes vs no)	0.000	2.32 (1.72–3.14)	0.039	2.45 (1.05–5.75)	0.112	1.37 (0.93–2.02)
Hypertension (yes vs no)	0.000	1.59 (1.23–2.06)	0.017	1.53 (1.08–2.17)	0.032	1.57 (1.04–2.37)
Retinopathy (yes vs no)	0.041	1.40 (1.01–1.92)	0.500	1.20 (0.70–2.06)	0.002	2.00 (1.30–3.07)
DKD history (yes vs no)	0.000	22.15 (14.72–33.31)	0.000	14.24 (7.55–26.86)	0.000	34.34 (19.78–59.62)
*A ratio: Carbohydrate (%)*						
50–60		Reference		Reference		Reference
< 50	0.128	1.46 (0.90–2.36)	0.589	1.18 (0.65–2.16)	0.754	0.85 (0.32–2.29)
> 60	0.527	1.17 (0.72–1.90)	0.090	2.11 (0.89–4.98)	0.705	1.14 (0.58–2.25)
A ratio: Fat (%)						
27–35		Reference		Reference		Reference
< 27	0.115	0.69 (0.44–1.09)	0.040	0.43 (0.19–0.96)	0.651	0.87 (0.47–1.60)
> 35	0.364	0.80 (0.50–1.29)	0.602	0.85 (0.47–1.56)	0.818	0.91 (0.41–2.04)
A ratio: Protein (> 12% vs ≤ 12%)	0.622	1.08 (0.80–1.44)	0.869	0.97 (0.63–1.48)	0.117	0.67 (0.40–1.11)

Adjusted OR estimated from the Binary logistic regression model with all the above variables added and with enter selection. CKD: Chronic kidney disease; BMI: body mass index; BP: blood pressure; LDL-C: low density lipoprotein cholesterol; TG: triglycerides; A: actual

## Discussion

The prevalence of CKD in Beijing (39.2%) was much higher than in Taiyuan (22.4%) and similar to that of Shanghai (38.6%) for hospitalised T2DM patients with similar diabetic duration and gender distinction [4]. The prevalence of CKD in Taiyuan was even lower than that of Hong Kong clinic patients (31.6%) with a shorter diabetic duration. This calls for our attention [5] and further examination. The difference between the Beijing and Taiyuan results can be attributed to variations in the prevalence of albuminuria, which was at a much higher rate in Beijing (38.6%) than in Taiyuan (21.6%) and Hong Kong (22.0%) and may have changed over time [4, 5]. Albuminuria has been observed to be somewhat transient and/or reversible. From serial cross-sectional studies of the National Health and Nutrition Examination Surveys (NHANES) in America from 1988 through 2014, the overall prevalence of CKD in adults with diabetes did not change significantly. However, the prevalence of albuminuria decreased from 20.8 to 15.9% with lower HbA_1c_, BP, LDL-C and TG values, and a higher rate of ACEI/ARB and statin usage [22]. The high prevalence of DKD (53.5%) and retinopathy (49.8%) history in the Taiyuan CKD patients—with a high corresponding rate of dyslipidaemia therapy (76.5%) or ACEI/ARB (60.5%) and statin (70.0%) usage at baseline – would indicate a previous CKD diagnosis and comprehensive management in our population base prior to this analysis. The regression of albuminuria may have already occurred, leading to a lower prevalence of CKD in Taiyuan.

Like earlier reports [3, 8, 23, 24], the risk factors for CKD were the following (after adjusting for potential variables): male, diabetic duration, poor ABC control, hyperuricemia, lack of regular activity and exercise, inadequate diet and a history of hypertension, retinopathy and DKD.

The poor ABC control in Beijing and Taiyuan in this study was comparable to the findings in other Chinese studies [4, 6], suggesting that a large proportion of the population may be in need of better primary care management. However, since an abnormal urinary albumin excretion can regress, remain the same or progress [25], a reversal of albuminuria and lower CKD prevalence may in the future be possible by reaching these target values: HbA_1c_ < 7.0%, BP < 130/80 mmHg, LDL-C < 2.6 mmol/L and TG < 1.7 mmol/L. The ABC control in the Hong Kong study was accomplished in 46.7% of patients with a BP of < 130/80 mmHg and in 61.7% of patients with an HbA_1c_ of < 7.0% [5]. These targets would be attainable with intensive insulin treatment (69.3% vs. 67.5%) and a high-rate usage of statin (76.8% vs. 73.3%)—or with dyslipidaemia therapy (78.6% vs. 78.6%)—during follow-up treatment in Beijing and Taiyuan. The high-rate usage of SGLT-2i (55.4% vs. 23.0%) and ACEI/ARB (53.9% vs. 64.6%) in Beijing and Taiyuan would afford added protective effects for the renal system and promote the reversal of albuminuria as described in the NHANES [22] studies. Treatment with SGLT-2i can even bring about the rapid reduction of UACR, usually within 12 weeks [26].

According to the nutritional evaluation, there was a significant difference in the dietary structure between Beijing and Taiyuan, not only among the overall study population, but among the CKD participants as well. Individual recommendations were made, taking into account nutritional balance, ABC control, and CKD protective needs. The actual carbohydrate, fat and protein in calorie ratios for Beijing were 49.6%:35.4%:14.4% and 61.5%:27.8%:10.8% for Taiyuan. The recommended adjustments were as follows: 53.0%:30.0%:17.0% for Beijing and 55.0%:27.0%:18.0% for Taiyuan. Although CKD patients were not found to have an excessive intake of fat or protein, a fat energy ratio of < 27 percent was associated with a lower risk of CKD in the Beijing participants—after allowing for potential variable factors such as smoking, alcohol consumption, educational level, diabetic duration and ABC control. A previous study identified a similar protective effect of a low-fat diet. When total red meat consumption was replaced with a low-fat diet, the positive association with CKD incidence was reversed [9]. The food source was recorded in our study, but no detailed classification was supplied, so no distinctions between total red meat, processed meat, or plant protein were drawn. A high-fat diet, on the other hand, was frequently accompanied by a high-calorie intake, increasing LDL-C and TG levels and leading to an impairment of kidney function in just eight weeks [27]. Thus, the protective effects of a low-fat diet may indeed exist, and this causal relationship must be further investigated in the future.

There are several limitations to this study. First, the testing equipment in each of the two hospitals was dissimilar, which would tend to impact the results in some ways. However, the research findings were still meaningful in terms of the sample size. Second, positive dietary effects on CKD prevalence may be due to a general increase in health awareness and individual compliance, and not exclusively due to a low-fat diet. The results do not allow the derivation of temporal relationships from a one-time assessment of eGFR, UACR, diet and the cross-sectional study design. Third, vinegar have been suggested ameliorating the hyperoxaluria-induced renal injury by improving the gut microbiota, insulin sensibibity and metabolomic profiles [28, 29]. Taiyuan people like to eat vinegar, while the consumtion and effect of vinegar were not discussed and might need further investigation in the future.

## Conclusion

In summary, this study focused on the prevalence and management of CKD in hospitalised patients with T2DM within two districts—Beijing and Taiyuan. Variations in the frequency of albuminuria—as well as higher rate of ACEI/ARB, SGLT-2i and dyslipidaemia usage—may be among the reasons for the differences in the prevalence of CKD in Beijing and Taiyuan. A future management direction can be clearly seen here. To prevent the onset and progression of CKD in patients with T2DM, a timely screening, a comprehensive risk factor control and the early use of renal protective medications are essential.

## Supplementary Information

**Additional file 1: Table S1.** The prevalence of CKD in Beijing and Taiyuan. eGFR: estimated glomerular filtration rate; eGFR categories: G1, eGFR ≥ 90.0; G2, eGFR 60.0–89.9; G3a, eGFR 45.0–59.9; G3b, eGFR 30.0–44.9; UACR Categories: A1 < 30.0, A2:30.0–300.0, A3 > 300.0; UACR: urinary albumin-to-creatinine ratio.** Table S2**. Dietary assessment and recommendations in Beijing and Taiyuan. A: actual; R: recommended; A/R: actual/recommended; R-A: recommended minus actual; IBW: ideal body weight. CKD: chronic kidney disease; Beijing vs. Taiyuan,* *P* < 0.05, # *P* < 0.001. Categorical variables were expressed as numbers. Continuous variables were expressed as medianfor non-normally distributed variables.

## Data Availability

The data that support the findings of this study are available from the corresponding author, LJM, upon reasonable request.
