# Supplementary material for: Differences in prevalence and management of chronic kidney disease among T2DM inpatients at the grassroots in Beijing and Taiyuan: a retrospective study
Source: J Health Popul Nutr. 2023 Jul 5;42:61. doi: 10.1186/s41043-023-00406-1 (PMC10320918; doi:10.1186/s41043-023-00406-1)
Supplement: Supplementary file 1 — Additional file 1: Table S1. The prevalence of CKD in Beijing and Taiyuan. eGFR: estimated glomerular filtration rate; eGFR categories: G1, eGFR ≥ 90.0; G2, eGFR 60.0–89.9; G3a, eGFR 45.0–59.9; G3b, eGFR 30.0–44.9; UACR Categories: A1 < 30.0, A2:30.0–300.0, A3 > 300.0; UACR: urinary albumin-to-creatinine ratio. Table S2. Dietary assessment and recommendations in Beijing and Taiyuan. A: actual; R: recommended; A/R: actual/recommended; R-A: recommended minus actual; IBW: ideal body weight. CKD: chronic kidney disease; Beijing vs. Taiyuan,* P < 0.05, # P < 0.001. Categorical variables were expressed as numbers. Continuous variables were expressed as medianfor non-normally distributed variables. [file 41043_2023_406_MOESM1_ESM.docx]

**Supplementary Table 1. The prevalence of CKD in Beijing and Taiyuan**

| **eGFR Categories** | **Beijing** | | | |  | **Taiyuan** | | | |
| --- | --- | --- | --- | --- | --- | --- | --- | --- | --- |
|  | **A1** | **A2** | **A3** | **Total** |  | **A1** | **A2** | **A3** | **Total** |
| G1 | 494 | 221 | 42 | 757 (88.2) |  | 713 | 138 | 17 | 868 (80.0) |
| G2 | 28 | 28 | 20 | 76 (8.9) |  | 129 | 47 | 13 | 189 (17.4) |
| G3a | 4 | 7 | 3 | 14 (1.6) |  | 9 | 5 | 6 | 20 (1.8) |
| G3b | 1 | 4 | 6 | 11 (1.3) |  | 0 | 3 | 5 | 8 (0.8) |
| Total | 527 (61.4) | 260 (30.3) | 71 (8.3) | 858 (100.0) |  | 851 (78.4) | 193 (17.8) | 41 (3.8) | 1085 (100.0) |

eGFR: estimated glomerular filtration rate; eGFR categories (mL/min/1.73m^2^): G1, eGFR≥ 90.0; G2, eGFR 60.0–89.9; G3a, eGFR 45.0–59.9; G3b, eGFR 30.0–44.9; UACR Categories (mg/g): A1<30.0, A2:30.0–300.0, A3＞300.0; UACR: urinary albumin-to-creatinine ratio.

Group 1: T2DM and CKD Group 1: T2DM only

**Supplementary table 2. Dietary assessment and recommendations in Beijing and Taiyuan**

| **Parameters** | **Beijig** | | | |  | **Taiyuan** | | | |
| --- | --- | --- | --- | --- | --- | --- | --- | --- | --- |
|  | **Overall (n=858)** | **Group 1 (n=336)** | **Group 2 (n=522)** | ***P*** |  | **Overall (n=1085)** | **Group 1 (n=243)** | **Group 2 (n=842)** | ***P*** |
| A:Total calories (kcal) | 1699 (1347, 2126) | 1689 (1347, 2163) | 1710 (1348, 2098) | 0.732 |  | 1784 (1513, 2110) # | 1785 (1530, 2105) | 1784 (1509, 2114) | 0.903 |
| R:Total calories (kcal) | 1590 (1442, 1742) | 1590 (1456, 1755) | 1582 (1427, 1731) | 0.147 |  | 1717 (1569, 1884) # | 1710 (1566, 1908) # | 1718 (1570, 1864) | 0.726 |
| A/R calories (%) | 106.2 (90.2, 127.8) | 105.8 (90.5, 127.0) | 106.6 (90.2, 128.4) | 0.886 |  | 104.5 (89.0, 123.2) | 105.1 (87.5, 122.4) | 104.4 (89.4, 123.2) | 0.822 |
| A/R Carbohydrate (%) | 97.0 (79.2, 116.2) | 96.6 (77.1, 115.7) | 97.4 (80.1, 117.0) | 0.408 |  | 116.2 (95.0, 144.0) # | 112.4 (93.4, 140.7) # | 117.0 (95.7, 145.3) | 0.242 |
| A/R Fat (%) | 123.3 (92.5, 164.3) | 123.3 (92.3, 166.1) | 123.2 (94.1, 162.8) | 0.952 |  | 103.4 (79.6, 126.6) # | 96.3 (74.1, 122.0) # | 104.8 (81.6, 128.1) | 0.007 |
| A/R Protein (%) | 92.1 (70.7, 115.3) | 91.0 (72.9, 121.3) | 92.6 (69.9, 113.3) | 0.443 |  | 64.6 (52.3, 84.1) # | 85.5 (59.9, 112.2) # | 61.9 (50.7, 76.6) | <0.001 |
| A ratio: Carbohydrate (%) | 49.6 (41.3, 56.3) | 49.0 (41.0, 56.4) | 49.8 (41.6, 56.3) | 0.640 |  | 61.5 (55.6, 67.9) # | 62.5 (56.4, 67.8) # | 61.0 (55.4, 68.1) | 0.158 |
| <50 | 445 (51.9) | 179 (53.3) | 266 (51.0) | 0.801 |  | 119 (11.0) # | 20 (8.2) # | 99 (11.8) | 0.092 |
| 50-60 | 285 (33.2) | 108 (32.1) | 177 (33.9) |  |  | 349 (32.2) | 71 (29.2) | 278 (33.0) |  |
| >60 | 128 (14.9) | 49 (14.6) | 79 (15.1) |  |  | 617 (56.9) | 152 (62.6) | 465 (55.2) |  |
| A ratio: Fat (%) | 35.4 (29.6, 41.5) | 35.7 (29.6, 42.1) | 35.2 (29.6, 40.9) | 0.538 |  | 27.8 (21.6, 32.9) # | 26.7 (21.1, 31.8) # | 27.9 (21.8, 33.4) | 0.131 |
| <27 | 136 (15.9) | 46 (13.7) | 90 (17.2) | 0.378 |  | 507 (46.7) # | 126 (51.9) # | 381 (45.2) | 0.109 |
| 27-35 | 277 (32.3) | 112 (33.3) | 165 (31.6) |  |  | 375 (34.6) | 81 (33.3) | 294 (34.9) |  |
| >35 | 445 (51.9) | 178 (53.0) | 267 (51.1) |  |  | 203 (18.7) | 36 (14.8) | 167 (19.8) |  |
| A ratio: Protein (%) | 14.4 (12.3, 16.6) | 14.3 (12.2, 16.5) | 14.6 (12.4, 16.7) | 0.322 |  | 10.8 (9.5, 12.3) # | 10.7 (9.5, 12.1) # | 10.8 (9.4, 12.3) | 0.695 |
| ≤12 | 194 (22.6) | 80 (23.8) | 114 (21.8) | 0.501 |  | 784 (72.3) # | 182 (74.9) # | 602 (71.5) | 0.297 |
| >12 | 664 (77.4) | 256 (76.2) | 408 (78.2) |  |  | 301 (27.7) | 61 (25.1) | 240 (28.5) |  |
| R ratio: Carbohydrate (%) | 53.0 (52.0, 54.0) | 53.0 (52.0, 55.0) | 53.0 (52.0, 54.0) | 0.036 |  | 55.0 (55.0, 55.0) # | 59.0 (55.0, 59.0) # | 55.0 (55.0, 55.0) | <0.001 |
| R ratio: Fat (%) | 30.0 (30.0, 30.0) | 30.0 (30.0, 30.0) | 30.0 (30.0, 30.0) | 0.469 |  | 27.0 (27.0, 30.0) | 30.0 (27.0, 30.0) # | 27.0 (27.0, 27.0) | <0.001 |
| R ratio: Protein (%) | 17.0 (16.0, 18.0) | 17.0 (16.0, 18.0) | 17.0 (16.0, 18.0) | <0.001 |  | 18.0 (18.0, 18.0) # | 11.0 (11.0, 18.0) # | 18.0 (18.0, 18.0) | <0.001 |
| IBW-A protein (g/kg) | 1.0 (0.8, 1.3) | 1.0 (0.8, 1.3) | 1.0 (0.8, 1.3) | 0.782 |  | 0.8 (0.7, 1.0) # | 0.8 (0.6, 1.0) # | 0.8 (0.7, 1.0) | 0.514 |
| <0.8 | 211 (24.6) | 88 (26.2) | 123 (23.6) | 0.657 |  | 504 (46.5) # | 123 (50.6) # | 381 (45.2) | 0.194 |
| 0.8-1.0 | 233 (27.2) | 91 (27.1) | 142 (27.2) |  |  | 335 (30.9) | 64 (26.3) | 271 (32.2) |  |
| >1.0 | 414 (48.3) | 157 (46.7) | 257 (49.2) |  |  | 246 (22.7) | 56 (23.0) | 190 (22.6) |  |
| IBW-R protein (g/kg) | 1.1 (1.0, 1.2) | 1.1 (1.0, 1.2) | 1.1 (1.0, 1.2) | 0.002 |  | 1.3 (1.2, 1.4) # | 0.8 (0.8, 1.3) # | 1.3 (1.3, 1.4) | <0.001 |
| <0.8 | 4 (0.5) | 4 (1.2) | 0 (0.0) | <0.001 |  | 55 (5.1) # | 55 (22.6) # | 0 (0.0) | <0.001 |
| 0.8-1.0 | 251 (29.3) | 124 (36.9) | 127 (24.3) |  |  | 94 (8.7) | 90 (37.0) | 4 (0.5) |  |
| >1.0 | 603 (70.3) | 208 (61.9) | 395 (75.7) |  |  | 936 (86.3) | 98 (40.3) | 838 (99.5) |  |

A: actual; R: recommended; A/R: actual/recommended; R-A: recommended minus actual; IBW: ideal body weight. CKD: chronic kidney disease; Beijing vs. Taiyuan,* P<0.05, # P<0.001. Categorical variables were expressed as numbers (%). Continuous variables were expressed as median (25% and 75% quartile) for non-normally distributed variables.

Group 1: T2DM and CKD Group 1: T2DM only
